# Evolution of parasitism along convergent lines: from ecology to genomics

**DOI:** 10.1017/S0031182013001674

**Published:** 2013-11-11

**Authors:** ROBERT POULIN, HASEEB S. RANDHAWA

**Affiliations:** 1 Department of Zoology, University of Otago, P.O. Box 56, Dunedin, New Zealand; 2 Ecology Degree Programme, Department of Botany, University of Otago, P.O. Box 56, Dunedin, New Zealand

**Keywords:** adaptive peaks, analogous traits, convergence, shared selective pressures, genome size, loss of functions

## Abstract

From hundreds of independent transitions from a free-living existence to a parasitic mode of life, separate parasite lineages have converged over evolutionary time to share traits and exploit their hosts in similar ways. Here, we first summarize the evidence that, at a phenotypic level, eukaryotic parasite lineages have all converged toward only six general parasitic strategies: parasitoid, parasitic castrator, directly transmitted parasite, trophically transmitted parasite, vector-transmitted parasite or micropredator. We argue that these strategies represent adaptive peaks, with the similarities among unrelated taxa within any strategy extending to all basic aspects of host exploitation and transmission among hosts and transcending phylogenetic boundaries. Then, we extend our examination of convergent patterns by looking at the evolution of parasite genomes. Despite the limited taxonomic coverage of sequenced parasite genomes currently available, we find some evidence of parallel evolution among unrelated parasite taxa with respect to genome reduction or compaction, and gene losses or gains. Matching such changes in parasite genomes with the broad phenotypic traits that define the convergence of parasites toward only six strategies of host exploitation is not possible at present. Nevertheless, as more parasite genomes become available, we may be able to detect clear trends in the evolution of parasitic genome architectures representing true convergent adaptive peaks, the genomic equivalents of the phenotypic strategies used by all parasites.

## INTRODUCTION

Transitions from a free-living existence to a parasitic one probably outnumber any other type of major evolutionary shift in life history strategy. Adoption of a parasitic mode of life has occurred repeatedly and independently more than once in many groups. For example, extant species of parasitic nematodes originate from several distinct transitions to parasitism (Blaxter *et al*. 1998). The same is true for many other taxa, such as copepods or isopods (see Poulin, 2011). Among red algae alone, there have been over 100 separate switches to a parasitic existence (Blouin and Lane, 2012). Thus, considering all eukaryotes, there have been several hundred independent transitions to parasitism over evolutionary time. Yet, despite their diverse origins and whether they have undergone extensive diversification or not, once they adopt parasitism as their mode of life, all these different lineages face the same set of selective pressures. They must all solve similar problems associated with host-to-host transmission, invasion of and survival within the host, and sustainable exploitation of host resources. There are only so many ways to achieve this successfully, and phylogenetically unrelated parasite lineages have therefore inevitably converged toward similar end-points in terms of their basic mode of parasitism (Poulin, 2011). Only a limited set of trait combinations can allow the persistence of a parasite population in both the short and long terms, and thus natural selection has pushed unrelated lineages down shared evolutionary paths toward one of these combinations.

The shared transmission and host exploitation strategies characterizing unrelated parasites are the outcome of convergent evolution, one of the most pervasive patterns seen in nature. Although many combinations of traits are theoretically possible, most are maladaptive, and selection forces distinct lineages to converge on those few viable combinations that yield higher fitness. These represent the peaks on Wright's (1984) adaptive landscape: combinations of traits that confer high fitness and toward which all evolutionary trajectories eventually lead. This, however, applies to the organisms’ phenotype, and does not require a parallel convergence at the genomic level. Convergent evolution toward similar phenotypes may happen simply because genetic and developmental constraints limit the number of possible phenotypes (Orr, 2005). In contrast, parallel evolution may be truly adaptive though different lineages attain analogous phenotypes via different genetic changes (Arendt and Reznick, 2008). Roughly identical traits in unrelated organisms may be the product of different cellular and physiological processes during development, and be the ultimate expressions of completely different genetic architectures. Therefore, the convergent evolution of parasites at the phenotypic level is not necessarily reflected at the genomic level.

In this brief review, we will first discuss the phenotypic convergence of eukaryotic parasite lineages toward only six general parasitic strategies. We will show that the similarities extend to all fundamental aspects of parasite exploitation of hosts, transmission among hosts, and even population-level parameters, transcending phylogenetic boundaries. Then, we will extend this discussion to general trends in the evolution of parasite genomes. This will be achieved by summarizing the findings of studies that have either compared the genomes of parasites with those of their closest free-living relatives in search of differences, or compared the genomes of unrelated parasite taxa in search of similarities. Other contributions to this Supplement of *Parasitology* will provide a much more detailed and in-depth examination of parasite genome evolution; here, we are only aiming at finding broad trends, if any, and comparing them to the evolutionary trends seen at the phenotypic level.

## CONVERGENCE IN PHENOTYPE AND ECOLOGY

Many biologists would say that the most obvious form of phenotypic convergence among parasite lineages is the widespread loss of morphological structures seen in many of them. The long-held view that parasites are morphologically simplified versions of their free-living relatives has now been disproven by the discoveries of novel sensory organs and other derived structures (Rohde, 1989; Brooks and McLennan, 1993). Although certain parasite taxa are indeed extremely simplified morphologically (e.g. Canning and Okamura, 2004; Noto and Endoh, 2004), in most other taxa the evolution of new sensory, feeding and attachment structures more than makes up for the loss of ancestral organs. The real convergence among parasite lineages occurred at a deeper functional and ecological level, where we can distinguish a limited number of strategies adopted independently by unrelated parasites.

Fitting disparate parasite species into broad categories based on common features inevitably requires sweeping potentially important differences under the rug. However, uncovering general patterns and processes demands that we temporarily ignore idiosyncrasies. The original categorization of parasites into microparasites and macroparasites has been influential for the development of epidemiological theory (Anderson and May, 1979; May and Anderson, 1979). The essential distinction is that the virulence of microparasites is not dependent on the number of separate infection events (microparasites multiply directly within the host), whereas that of macroparasites is proportional to the number of individual parasites that infect the host, i.e. it is intensity-dependent. Later, another categorization of parasites used a series of dichotomies in life cycle complexity and virulence patterns to split parasites into several strategies representing convergent evolutionary trajectories (Kuris and Lafferty, 2000; Lafferty and Kuris, 2002). More recently, Poulin (2011) proposed a modification of this classification to base parasite strategies solely on convergent properties and not on those determined by phylogenetic history. The six strategies of eukaryotic parasites proposed by Poulin (2011) correspond to the finite set of trait combinations that represent the only adaptive peaks in the parasite evolutionary landscape. Their main characteristics are summarized in Table 1, and they are briefly described here.

**Table 1.** Main life history and ecological traits characterizing the six strategies on which eukaryote parasites have converged

	Parasitoids	Parasitic castrators	Directly transmitted parasites	Trophically transmitted parasites	Vector-transmitted parasites	Micropredators
Life cycle	1 host from 1 species	1 host from 1 species	1 host from 1 species	1 host from ⩾2 species	1 host from ⩾2 species	>1 host from ⩾1 species
Virulence	Maximum	Maximum	Generally low; intensity-dependent	Generally high in IH, low in DH; intensity-dependent	Generally high in host, moderate in vector	Generally low; intensity-dependent
Host manipulation	Common	Common?	No	Common (in IH)	Common (in vector)	No
Parasite:host size ratio	From 1:10^2^ to 1:1	From 1:10^2^ to 1:1	Variable	Variable	From 1:10^8^ to 1:10^6^	Variable
Prevalence	Low	Low	Variable	Variable; often low in IH, moderate to very high in DH	Variable	N/A
Mean intensity of infection	Low (close to 1/host)	Low (close to 1/host)	Variable	Variable; often low to moderate in IH, moderate to very high in DH	Variable; usually low to moderate in both host and vector	N/A

IH = intermediate host; DH = definitive host; virulence = loss of host fitness.

### Parasitoids

These are parasites that grow to relatively large sizes within their host and almost inevitably kill it when they complete their development and emerge from the host. They include hymenopteran and dipteran insects, nematomorphs, mermithid nematodes, oenonid polychaetes and *Cordyceps* fungi. Remarkably, the ability to alter host behaviour markedly around the time of parasite emergence, in ways that benefit the parasite, has evolved independently in multiple lineages of parasitoids (e.g. Maitland, 1994; Eberhard, 2000; Thomas *et al*. 2002; Poulin and Latham, 2002; Grosman *et al*. 2008). As their large size and high virulence would not allow the long-term persistence of species with widespread infections throughout a host population, only species with life history traits that lead to a sustainable exploitation of host resources have persisted over evolutionary time; therefore, parasitoids typically occur at low prevalence and low mean intensity of infection (close to a single parasite per infected host).

### Parasitic castrators

These parasites induce the total or near-total suppression of host reproduction early in the infection, and use the resources that the host would have invested in its reproduction for their own reproduction. This is their main difference from parasitoids, which they resemble in many other aspects. Indeed, castrators share many traits with parasitoids (Kuris, 1974): they also attain relatively large body sizes, and occur at low prevalence and low intensities of infection in their host populations. Parasitic castrators include taxa such as ascothoracican and rhizocephalan barnacles, entoniscid isopods, strepsipteran insects, and the juvenile stages of some helminths, most notably the intramolluscan stages of trematodes.

### Directly transmitted parasites

Parasites using this strategy require a single host individual per generation, in which they induce variable but intensity-dependent pathology. Directly transmitted parasites include copepods, cyamid amphipods, lice, mites, monogeneans, many nematodes, many fungi and many taxa of protists (*Eimeria, Giardia*); many bacteria and viruses would also fall within this category. The infection mode used by these various taxa to get inside the host or attach to the host's outside surfaces vary considerably, but these mechanistic differences do not detract from the more fundamental life history similarities uniting these parasites. Certain parasites present unusual features, but nevertheless fit within this strategy. For instance, the many single-celled parasites capable of within-host multiplication, and entomopathogenic nematodes which multiply within their host after the latter is killed by symbiotic bacteria carried by the nematodes, are still fundamentally directly transmitted parasites. At the population level, directly transmitted parasites can display a range of prevalence and mean intensity values, but are almost invariably characterized by an aggregated distribution in which most individual hosts harbour few or no parasites, whereas a few hosts harbour the majority of the parasite population (Shaw and Dobson, 1995; Poulin, 2007, 2013). Direct transmission is probably the first strategy adopted by any taxon following its transition to a parasitic mode of life; later, during the course of evolution, extra hosts can be added to the life cycle and other fundamental traits may change, leading to the adoption of any of the other, derived strategies.

### Trophically transmitted parasites

These parasites require two or more hosts of different species, in a particular order, to complete a single generation, and are acquired by their definitive host (the one in which they mature and reproduce; almost always a vertebrate) via predation on the preceding host. Trophically transmitted parasites include all trematodes (except schistosomes, in which trophic transmission has been secondarily lost) and cestodes, acanthocephalans, pentastomids, numerous nematodes and many taxa of protists (e.g. *Toxoplasma, Sarcocystis*). The distribution of trophically transmitted parasites among their hosts is characterized by aggregation (Poulin, 2013), just like that of directly transmitted parasites. They can be quite virulent to their intermediate hosts. However, the most notable effect on the intermediate host is the ability of many trophically transmitted parasites to manipulate the phenotype of the intermediate host in ways that increase its susceptibility to predation by the definitive host (Moore, 2002; Poulin, 2010). Some of these manipulations result in spectacular alterations in the behaviour or appearance of the intermediate host that are much too specific and targeted to be the products of chance. This adaptive trait has evolved independently in numerous lineages of trophically transmitted parasites, indicating that it is part of the converging suite of core traits characterizing this parasitic strategy.

### Vector-transmitted parasites

These parasites require two hosts of different species to complete one generation: a vertebrate host and a micropredator (see below) that serves as a vector transporting the new generation of parasites from its host of origin to a new host. Vector-transmitted parasites include filaroid nematodes and numerous taxa of protists (*Plasmodium, Babesia, Leishmania, Trypanosoma*, etc.); of course, numerous fungi, bacteria and viruses also are vector-transmitted. Parasites using this strategy are invariably small, both in absolute terms and relative to their host, and potentially highly virulent through replication within the host. In addition, manipulation of vector behaviour to increase transmission efficiency has evolved independently in several lineages of vector-transmitted parasites (Moore, 1993), adding another typical feature to this strategy.

### Micropredators

Parasites using this strategy use an indeterminate number of host individuals (of the same or different species) per generation. Each association with a host is brief, lasting from seconds to days depending on the species, and is followed by a period spent off the host moulting or laying eggs. Most micropredators feed on host blood, and include taxa such as leeches, branchiuran crustaceans, gnathiid isopods, blood-sucking dipteran insects (mosquitoes, sand flies, etc.), fleas, ticks, lampreys and vampire bats. Unless they are large relative to their host (e.g. a lamprey feeding on a medium-sized fish), their direct impact on host fitness is generally minimal; however, by vectoring virulent pathogens (see above), their indirect impact can be severe.

The categorization of parasites into the above six strategies requires only a consideration of how many hosts they require per generation, and of what they do to these hosts. Of course, on more detailed physiological or anatomical levels, evidence of convergence is also easy to find. For example, many directly transmitted and most trophically transmitted parasites inhabit the vertebrate gut. As a consequence, phylogenetically unrelated parasites that have adopted these strategies display similar adaptations to an anaerobic environment and similar defences against enzymatic attack. Similarly, numerous unrelated taxa have independently evolved multi-host life cycles by adding a predator (upward incorporation) or a prey (downward incorporation) of the original host to exploit both participants of a predator–prey interaction (Parker *et al*. 2003). Many such lineages that converged toward the trophically transmitted parasite strategy have since independently evolved other strikingly similar adaptations to their life cycle, including manipulation of the intermediate host (see above), asexual multiplication in the intermediate host (Moore and Brooks, 1987; Galaktionov and Dobrovolskij, 2003), and facultative truncation of the life cycle (Poulin and Cribb, 2002; Levsen and Jakobsen, 2002; Andreassen *et al*. 2004; Lefebvre and Poulin, 2005).

Perhaps the most convincing line of evidence for the universality of these six strategies as end points of evolution comes from the fact that they also capture the range of host exploitation strategies seen among parasites of plants. As hosts, plants are in many ways very different from animals. Their lower metabolic rate means that less energy can be extracted from them per gram of tissue or per unit time than from an animal. Plants have tough cell walls and thick external surfaces, with no easy access to their internal tissues comparable to an animal's mouth. Yet, with the exception of trophically transmitted parasites (because plants are autotrophic), all other parasite strategies are represented among organisms exploiting plants as their hosts (see Poulin, 2011). Among arthropods feeding on plants, there are micropredators that take small meals of sap from many plants during their lifetime (e.g. leafhoppers). These often carry vector-transmitted parasites from plant to plant, including pathogenic fungi, bacteria and viruses (Agrios, 1997). Other arthropods, like scale insects, have a strategy identical to that of directly transmitted parasites (Edwards and Wratten, 1980). And yet others can be seen as parasitoids. Consider a brood of caterpillars hatching on a small shrub: as they increase in size and before they pupate (equivalent to emerging from the host), they can consume a large amount of leaf tissue and cause the photosynthetic death of the plant host. To these arthropod parasites, we could add fungi that behave as castrators (Roy, 1993), and plant-parasitic nematodes which are essentially all directly transmitted parasites (Jasmer *et al*. 2003). Finally, parasitic angiosperms (flowering plants) that derive sustenance from other plants can also be categorized into the strategies defined above for animal parasites. Most, like mistletoes and dodder, have opted for a strategy superficially different from but fundamentally identical to that of directly transmitted parasites (Press and Graves, 1995; Poulin, 2011). Some, like strangler figs, are true parasitoids (Putz and Holbrook, 1989).

Just as the evolution of parasitism appears inevitable, having occurred repeatedly and independently in all major lineages of unicellular and multicellular organisms, their subsequent convergence toward one of the above strategies also seems inescapable (see Poulin, 2011, for further evidence and discussion). These adaptive strategies consist of particular combinations of traits that have survived the selection process, while lineages adopting different trait combinations have gone extinct. In the next section, we look for comparable general or convergent trends in the evolution of parasite genomes.

## TRENDS IN GENOMIC EVOLUTION

Many of the early views on the evolution of parasite genomes seem to reflect the widespread, almost dogmatic notion that parasites are retrogressive and evolutionary degenerates: the loss of morphological structures is expected to be paralleled by a loss of genetic diversity (Frank *et al*. 2002). As mentioned earlier, the apparent loss of morphological structures seen in many parasite lineages has been compensated by new structures or adaptations tailored to a parasitic existence (Brooks and McLennan, 1993). Therefore, general morphological evolution should not blindly be viewed as a signature of corresponding evolutionary changes in the genome. Along the same lines, the strong convergence seen among parasite taxa at the phenotypic level is not necessarily aligned with corresponding genomic changes seen across taxa. Analogous traits evolving independently in different lineages, based on different genetic pathways, can still yield similar phenotypes and allow different lineages to meet on the same adaptive peak.

In what follows we summarize the broad, general trends in the genomic evolution of parasites. We do this for different aspects of genomic organization, and use published comparisons of the genomes of parasites with those of their closest free-living relatives to find differences, or comparisons of the genomes of unrelated parasite taxa to seek similarities. Given the relatively few studies available to date, we adopt a broad taxonomic coverage that is fully compatible with our goal of seeking generalities. However, despite increased sequencing efforts in recent years, the bulk of sequenced parasite genomes remain restricted primarily to species of medical, veterinary and economic relevance. Consequently, generalities highlighted here are likely biased and are based on limited coverage within each taxonomic group. However, genomic comparisons between parasites and their closest free-living relatives reveal some trends that could form the basis for future ecological and evolutionary testable hypotheses.

### Genomic reduction and compaction

Parasitism has evolved independently on multiple occasions within different taxonomic groups (Blaxter *et al*. 1998; Poulin, 2011; Blouin and Lane, 2012). Thus far, genome-mining has yet to uncover a single universal gene, or subset of genes, associated with parasitism (Rödelsperger *et al*. 2013). Rather, selective pressures associated with parasitism, such as metabolic and spatial economy and cell multiplication speed (Cavalier-Smith, 2005), have placed similar evolutionary constraints on genomes of parasitic organisms leading to a convergence towards genome reduction or compaction. Although not universal (e.g. Kikuchi *et al*. 2011; Raffaele and Kamoun, 2012), nuclear genome reduction in parasitic organisms (compared with their free-living relatives) has been observed in a wide range of taxa, including amoebozoans (Glöckner and Noegel, 2013), bacteria (Touchon *et al*. 2011), fungi (Cushion, 2004; Heinz *et al*. 2012), mites (Mounsey *et al*. 2012), nematodes (Kikuchi *et al*. 2011; Rödelsperger *et al*. 2013) and parasitoid hymenopterans (Ardila-Garcia *et al*. 2010). The most extreme examples of genome reduction occur in intracellular parasites. For instance, the microsporidian *Encephalitozoon intestinalis* has the smallest known eukaryotic nuclear genome at 2·3 Mb (Corradi *et al*. 2010), which is much smaller than that of many free-living bacteria (Corradi and Slamovits, 2011). Although no complete nuclear genome for free-living relatives of the Platyhelminthes has been sequenced and annotated, the 106–147 Mb nuclear genomes of tapeworms (Olson *et al*. 2012; Tsai *et al*. 2013) are smaller than that of the free-living planarian *Schmidtea* (700 Mb) (see Olson *et al*. 2012) and those of the parasitic trematodes *Schistosoma mansoni* (363 Mb) and *Clonorchis sinensis* (516 Mb) (Berriman *et al*. 2009 and Wang *et al*. 2011, respectively). Furthermore, based on a comparison of C-values, or amount of DNA within a haploid nucleus, available in the Animal Genome Size Database, those of trematodes and cestodes are generally lower than those reported for free-living planarian flatworms (Gregory, 2013) and suggest nuclear genome reduction in parasitic flatworms consistent with most other taxa.

In addition, convergent trends of genomic reductions associated with parasitism are not limited to the nucleus. Organelle genome reduction is an evolutionary trend common in many parasitic organisms (Rocha and Danchin, 2002; Keeling and Slamovits, 2005; Cafasso and Chinali, 2012). For instance, mitochondria are either absent or retained as mitochondria-derived organelles (e.g. mitosomes) in a variety of taxa including apicomplexans (Corradi and Slamovits, 2011; Hikosaka *et al*. 2013), microsporidians (Katinka *et al*. 2001) and amoebozoans (Glöckner and Noegel, 2013) or reduced in size such as in ciliates (Burger *et al*. 2000), red algae (Hancock *et al*. 2011), trematodes and cestodes (Le *et al*. 2002), and nematodes (Hu *et al*. 2003). Nevertheless, mitochondrial genome sizes in one family (Mermithidae) of nematodes parasitic in insects far exceed those reported for free-living nematodes, an exception to the general trend with no clear explanation (Lagisz *et al*. 2013).

Should genomic reductions, whether nuclear or organellar, result in a loss of genetic information? Not necessarily. Genome compaction is generally achieved through a reduction in the number of mobile or repetitive genetic elements (e.g. Kissinger and de Barry, 2011; Olson *et al*. 2012), protein shortening (e.g. Katinka *et al*. 2001) or reduced intergenic spacers and introns (e.g. Keeling and Slamovits, 2005). Generally, in parasitic organisms, genome compaction has led to an increase in gene density compared with their free-living relatives (e.g. Keeling and Slamovits, 2005; Rödelsperger *et al*. 2013).

### Functional loss

As a result of evolving alongside their respective hosts and relying on the latter to provide shelter and resources, parasitic organisms from a wide range of taxa have either lost or simplified/reduced several metabolic pathways (Müller *et al*. 2012). For instance, many parasitic plant species lack chlorophyll and have lost their photosynthetic ability (de Pamphilis and Palmer, 1990; Revill *et al*. 2005), the apicomplexan *Cryptosporidium parvum* has lost the ability to synthesize nucleotides *de novo* (Striepen *et al*. 2004) and cestodes are unable to synthesize cholesterol *de novo* (Olson *et al*. 2012). In other taxa, the loss of function is incomplete such as in some microsporidians, which have lost all but two of 21 genes involved in glycolysis (Keeling *et al*. 2010). Furthermore, several groups of parasitic organisms having evolved in anaerobic environments, such as amoebozoans and microsporidians, have undergone a secondary reduction of the mitochondria (Müller *et al*. 2012), having lost nearly all mitochondrial function, including the ability to generate ATP (Heinz *et al*. 2012; Heinz and Lithgow, 2013 and references therein). On the other hand, many other groups of parasitic organisms have conserved 36 of 37 genes typically found in animal mitochondria (only lacking the subunit 8 of the adenosine triphosphatase complex), including nematodes (Hu *et al*. 2003; Kang *et al*. 2009; Liu *et al*. 2013), monogeneans (Kang *et al*. 2012), cestodes (Jeon *et al*. 2005, 2007; Kim *et al*. 2007; Park *et al*. 2007) and trematodes (Le *et al*. 2001*a*, *b*, 2002), whereas this subunit has been lost in the mitochondria and transferred to the nucleus in ciliates (Burger *et al*. 2000). Other losses appear unrelated to the parasitic mode of life *per se*, though they may be indirect consequences of other evolutionary changes associated with parasitism; for instance, cestodes have lost several homeobox gene families (Tsai *et al*. 2013), which may account for their simplified morphology. Overall, however, the generality associated with these functional losses is that selective pressures towards genome reduction have resulted independently in the loss of entire gene families or functions and in the reduction of metabolic pathways primarily associated with a free-living existence (Katinka *et al*. 2001; Corradi *et al*. 2010).

### Functional gains

Horizontal or lateral gene transfer (HGT) from prokaryotes to eukaryotes is identified as a process by which new genes of adaptive significance may have been acquired by parasitic organisms (Alsmark *et al*. 2013). Such transfers appear to have played a significant role in the evolution of virulence (Gardiner *et al*. 2012). For instance, it is believed that ancestral bacterivorous nematodes acquired cell wall-degrading enzymes from soil bacteria via HGT, thus gaining the ability to parasitize plants (Keen and Roberts, 1998; Abad *et al*. 2008; Paganini *et al*. 2012). For example, the plant parasite *Bursaphelenchus xylophilus* possesses glycoside hydrolase enzymes capable of breaking down the plant cell wall most likely acquired from bacteria or possibly fungi (Kikuchi *et al*. 2011). Pathogenicity in fungi is also hypothesized to have originated via HGT through the acquisition of secreted cell wall proteins (Butler *et al*. 2009). Other examples of genes acquired via HGT include enzymes involved in the nucleotide salvage pathway in *C. parvum* (Striepen *et al*. 2004) and ATP transporters in microsporidians (Pombert *et al*. 2012). Although the evolution of many novel genes in parasitic organisms has arisen via HGT, only those under positive selection have undergone extensive gene duplication and successfully integrated into their new respective genomes (Blaxter, 2007). Despite a common trend towards genome reduction, examples of selective expansion in the genomes of parasitic organisms are plentiful (e.g. Huang *et al*. 2004; Loftus *et al*. 2005). For instance, approximately 40% of genes in the *Giardia* genome have undergone gene duplication, with the majority of these involved in evading the host's immune response (Sun *et al*. 2010) and selective expansion of secreted cell wall proteins following HGT has been observed in parasitic fungi (Butler *et al*. 2009). However, some parasitic organisms have evolved ways of expanding certain gene families associated with evading their host's immune system while maintaining a highly compact genome. For instance, certain rickettsiales bacteria have pseudogenes that possess conserved 5′ and 3′ ends flanking a hypervariable region. When recombined into a functional expressed site, these pseudogenes generate new antigenic variants (Brayton *et al*. 2001). Also, antigenic diversity in *Plasmodium falciparum* is achieved by maintaining large, multicopy, and hypervariable gene families such as variant antigen repertoire (*var*) genes (Su *et al*. 1995). Throughout the genome of *P. falciparum*, approximately 60 *var* genes encode proteins composed of conserved basic architecture with hypervariable amino acid sequence translating into a potentially unlimited repertoire of membrane proteins (see Dzikowski *et al*. 2006). By encoding a single membrane protein at any given time, as the host mounts an immune response against this particular variant, sub-populations encoding different membrane proteins are selected for, leading to a proliferation of the parasitic infection (see Dzikowski *et al*. 2006). Furthermore, recent evidence suggests that many molecules involved in epigenetic memory, regulation of gene expression, and immune evasion in apicomplexans have been acquired via HGT from eukaryotes (Kishore *et al*. 2013).

Lineages that have adopted parasitism as a mode of life are under strong selective pressures at the genomic level, with potentially important evolutionary consequences. Genome reductions/compactions may provide a significant adaptive potential, which has allowed the evolution of an intracellular lifestyle or the exploitation of intermediate hosts orders of magnitude smaller than their definitive hosts. The loss of genes associated with processes/functions redundant with those carried out by the hosts and strong selection towards genomes that are A-T rich (Moran, 1995; Cavalier-Smith, 2005) are all means to preserve energy, whereas the gain of new genes via HGT and their establishment into the genomes has led to the evolutionary specialization of parasites. Although many of the processes leading to parasitism may have arisen by chance, they have evolved through positive selection. Overall, from a genomic perspective, parasites are highly efficient at what they do and are nothing like the overly simplified version of their free-living relatives often depicted in textbooks.

## CONCLUSION

Whether at the phenotypic or genomic level, evidence of convergent evolution is not hard to find among unrelated parasitic organisms. However, we are still a long way from matching particular changes in parasite genomes with most of the broad phenotypic traits that define the convergence of parasites toward only six strategies of host exploitation, corresponding to six stable adaptive peaks. For instance, no set of genes can currently be linked directly to whole-organism features such as the use of multiple hosts per generation, or the ability to manipulate a suite of host phenotypic traits to enhance transmission success. Coupling between gene and function is possible for more specific traits such as many metabolic pathways. In contrast, complex traits such as the nature of the life cycle and what effects a parasite has on host behaviour or fitness are emergent properties underpinned by a network of interacting genes, and thus simple links between genome and phenotype will not be so easy to identify. Nevertheless, cases of genome reduction or compaction and losses or gains of genes suggest parallel changes in the genomes of unrelated parasite taxa. The taxonomic coverage of parasites with fully sequenced genomes will expand in the next few years to include a broader range of species within the main parasitic groups, as well as thus far neglected groups such as nematomorphs and pentastomids. We may then be able to delineate a small number of clear trends in the evolution of parasitic genome architectures representing true convergent adaptive peaks, the genomic equivalents of the six phenotypic strategies seen among extant parasites.
